# Efficacy of Different Dietary Patterns in the Treatment of Functional Gastrointestinal Disorders in Children and Adolescents: A Systematic Review of Intervention Studies

**DOI:** 10.3390/nu15122708

**Published:** 2023-06-10

**Authors:** Christina N. Katsagoni, Vasiliki-Maria Karagianni, Alexandra Papadopoulou

**Affiliations:** 1 Department of Clinical Nutrition, Agia Sofia Children’s Hospital, 11527 Athens, Greece; christina.katsagoni@gmail.com; 2 Division of Gastroenterology and Hepatology, First Department of Pediatrics, University of Athens, Agia Sofia Children’s Hospital, 11527 Athens, Greece; v.m.karagianni@gmail.com

**Keywords:** functional gastrointestinal disorders, FGIDs, IBS, dyspepsia, constipation, abdominal pain, low-FODMAP diet, fructose- or lactose-restricted diet, gluten-free diet, Mediterranean diet

## Abstract

Functional gastrointestinal disorders (FGIDs) are common in children and adolescents. In recent years, interest in the role of diet in the treatment of FGIDs has increased. Currently, interest focuses on the low-FODMAP diet (LFD), the fructose- or lactose-restricted diet (FRD or LRD), the gluten-free diet (GFD), and the Mediterranean diet (MD). In this review, we focus on the role of these dietary patterns in the FGIDs most commonly diagnosed in clinical practice, namely irritable bowel syndrome (IBS), functional abdominal pain (FAP), functional dyspepsia (FD), and functional constipation (FC). Fifteen clinical trials were systematically reviewed (both RCTs and single-arm clinical trials). We demonstrated the lack of high-quality intervention trials. Based on current evidence, low-FODMAP diet, LRD, FRD, and GFD have no place in daily clinical practice for the management of children and adolescents with FGIDs. Nevertheless, some patients with IBS or RAP may experience some benefit from the use of a low-FODMAP diet or FRD/LRD. Limited data suggest that MD may be promising in the management of FGIDs, especially in IBS patients, but more data are required to investigate the mechanisms of its protective effects.

## 1. Introduction

Functional gastrointestinal disorders (FGIDs) are common in children and adolescents [1]. Since there are not biomarkers or specific tests to diagnose FGIDs, their diagnosis is based on symptom-based criteria [2]. The Rome IV criteria for children and adolescents [3,4] are the current criteria used to diagnose childhood FGIDs that replaced the previous published Rome III criteria [5,6]. Depending on the criteria used, the prevalence rates vary in childhood, ranging from 9.9% to 29% [7,8,9].

With regard to the pathogenetic mechanism of FGIDs, the literature provides new insights regarding a possibly multifactorial pathogenesis of FGIDs, although it remains elusive. A biopsychosocial (systems) model seems to better explain this notion, suggesting that early life factors may influence the clinical presentation of the disorder and clinical outcome [10]. Possible factors include genetic predisposition [11], altered gut–brain axis and gut motility [12], gut hypersensitivity [13], gut inflammation/infection [14], altered microbiome composition [15], psychological conditions [16], and environmental triggers such as food [10,17].

FGIDs are considered separate but overlapping diseases in both children and adults under the Rome IV criteria [18]. In children and adolescents, FGIDs result in a significant symptom burden [3], which is of public health concern, since they are associated with functional disability, reduced quality of life, anxiety, school absenteeism, parental work absenteeism, and a notable increase in health care costs [7].

Currently available treatment options include fiber supplementation [19], probiotics [20], cognitive behavioral therapy [21], psychosocial interventions [22], fecal microbiota transplantation [23], centrally and peripherally acting neuromodulators (such as antidepressants) [24], laxatives [25], antispasmodics, and prokinetics [26]. However, in recent years, there has been renewed interest in the role of specific dietary patterns in the treatment of FGIDs. Currently, interest is focused on the low-FODMAP diet [27], fructose- or lactose-restricted diet (FRD or LRD) [28], gluten-free diet (GFD) [29], and Mediterranean diet (MD) [30].

In the present systematic review, we provide an up-to-date overview of the efficacy of specific dietary patterns as treatment options in ameliorating functional gastrointestinal (GI) symptoms of the most commonly diagnosed FGIDs in clinical practice, namely IBS, functional abdominal pain (FAP), functional dyspepsia (FD), and functional constipation (FC), in children and adolescents aged 3 to 18 years old.

## 2. Materials and Methods

A systematic literature search was performed up to 1 April 2023, using specific keywords in the databases of US National Library of Medicine (PubMed.gov) and Scopus (www.scopus.com). Two independent researchers (C.N.K and V.-Μ.K.) identified all relevant publications. Studies were assessed using a hierarchical approach based on the title, the abstract, and finally, the full texts of the studies. The Medical Subject Heading (MeSH) keywords used were Rome III-IV criteria, FGIDs, IBS, functional abdominal pain [recurrent abdominal pain (RAP) or continuous (CAP)], functional dyspepsia, functional constipation, Mediterranean diet, low-FODMAP diet, gluten-free diet, fructose intolerance/malabsorption, lactose intolerance/malabsorption, fructose- and/or lactose-restricted diet, low-fructose diet, low-lactose diet, diet, nutrition, RCT, and clinical trial, as well as combinations of the above terms in children or adolescents. The reference list of the retrieved articles or reviews was used to search for other relevant studies. The Preferred Reporting Items for Systematic Review and Meta-Analyses (PRISMA) guidelines [31] were followed in the present study.

### 2.1. Inclusion and Exclusion Criteria

Inclusion criteria: Intervention studies, namely randomized (with crossover or parallel design) (RCTs) and non-randomized controlled clinical trials (non-RCTs) or single-arm trials were included. All studies were written in English assessing the effects of any of the corresponding types of diet, namely low-FODMAP diet, fructose- or lactose-restricted diet, GFD, and MD, on children and adolescents (aged 3 to 18 years old) with at least one FGID, i.e., IBS, FAP (RAP or CAP), FD, or FC. FGIDs diagnosis was in alignment with Rome III-IV criteria or other precise definition provided by the authors while excluding any known pre-existing GI symptoms or organic conditions.

Exclusion criteria: case–control, cross-sectional, or non-human studies; case-reports; studies in adult population; editorial; commentary; abstracts; review articles; and meta-analysis were excluded.

### 2.2. Data Extraction

The assessment of all relevant studies was conducted with the Rayyan web tool. Data from the eligible studies were independently and blindly extracted by two investigators (C.N.K. and A.P.) in duplicates. Any disagreements were resolved after discussion between investigators. For all studies, we extracted information on inclusion and exclusion criteria, authors, journal and year of publication, methods (study design), study sample, patient population characteristics (number, age, diagnostic criteria (if available), type of FGID), intervention (type, duration of intervention), dietary dosage (if available), control (number, description), follow-up, outcomes measured, tools used to measure the outcomes of each study, and study results.

### 2.3. Outcome Measured

The primary outcome from the included studies was the efficacy of a low-FODMAP diet, FRD or LRD, GFD or MD in improving abdominal pain (i.e., number, frequency, or severity of pain episodes, or another measure stated by the authors).

Secondary outcomes included changes in other GI symptoms (i.e., distension, gas production, vomiting, nausea), stool consistency, quality of life (QoL), interference in daily activities, and adherence to the intervention diet(s).

All measurements should have been defined by authors using a validated defined measurement tool.

### 2.4. Study Quality

The Risk Of Bias In Non-randomized Studies—of Interventions (ROBINS-I) tool [32] was used to assess the risk of bias of non-RCTs/single-arm clinical trials, based on the following domains: (1) bias due to confounding, (2) bias in selection of participants into the study, (3) bias in classification of interventions, (4) bias due to deviations from intended interventions, (5) bias due to missing data, (6) bias in measurement of the outcome, and (7) bias in selection of the reported results. The overall judgement of the quality of non-randomized clinical trials was based on the worst level of bias that each study received for a particular domain [33].

For RCTs, the Cochrane Risk of Bias tool (ROB2) [34] was used to assess their quality, based on the following criteria: (1) bias due to randomization process, (2) bias due to deviations from intended interventions, (3) bias due to missing data, (4) bias in measurement of the outcome, (5) bias in selection of the reported results. For RCTs with crossover design, the ROB2 for crossover trials was used [35].

The overall bias of the included studies was categorized as “low risk of bias” if all domains of the study were at low risk, “some concerns” if at least one domain of some concerns existed but no high-risk domains, and “high risk of bias” if at least one domain of the study was at high risk or multiple domains raised some concerns [34,35].

## 3. Results

**Figure 1:**
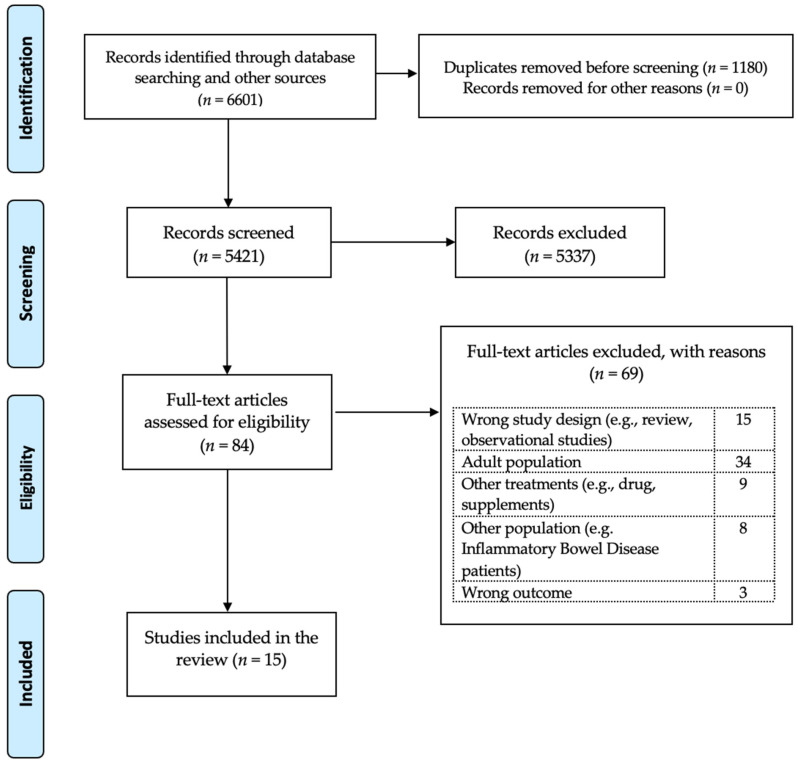
Flowchart of the.systematic review based on PRISMA guidelines.

In total, 84 full-text studies were assessed for eligibility. Of those, 15 clinical trials met the inclusion and exclusion criteria and were selected for the present systematic review. In specific, six studies evaluated the efficacy of a low-FODMAP diet [36,37,38,39,40,41,42], five evaluated the efficacy of FRD/LRD [28,43,44,45,46], three trials evaluated the efficacy of the GFD [47,48,49], and one evaluated the efficacy of the MD [50]. The flowchart of the eligible studies is shown in Figure 1.

Findings with regard to the quality of the eligible studies (i.e., nonRCTs/single-arm clinical trials, RCTs with a crossover design, and other RCTs) are shown in the Supplementary Materials, Figures S1–S3, accordingly. Based on the tools used, 3/15 studies showed “low” risk of bias, 8/15 showed moderate risk (i.e., raised “some concerns” in one or multiple domains), and 4/15 were characterized as having “serious” risk of bias. 

### 3.1. Low-FODMAP Diet

The diet that is low in fermentable oligosaccharides, disaccharides, monosaccharides, and polyols (FODMAPs) is a widely accepted approach for the management of IBS in adults [51], recommended also by the American College of Gastroenterology [52,53]. The main dietary sources of FODMAPs include certain fruit, vegetables, legumes, and artificial sweeteners [51]. The symptoms in some patients are mainly generated through two ways; (a) the unabsorbed fructose, polyols, and lactose increase small intestinal water content and therefore the intestinal motility and (b) the indigestible fructans and galacto-oligosaccharides undergo rapid microbial fermentation and cause increased gas production, flatulence, and abdominal distension [54].

Although numerous pooled data highlight the efficacy of this diet for the management of symptoms in adult patients with FGIDs [51,55,56], current data in pediatrics are insufficient, showing conflicting results [57].

In total, six relevant studies were found in pediatrics: three studies evaluated the effect of a low-FODMAP diet on GI outcomes in IBS patients [36,37,38], and three studies assessed its effect on FAP, FC, or FD [39,40,42]. Four studies were RCTs [36,38,40,42], and two studies were non-randomized clinical trials [37,39]. FGIDs diagnosis was based on Rome III criteria in four studies [36,37,39,40] and on Rome IV in two studies [38,42]. Only two studies reported the exact amount of FODMAPs given to the study participants [36,38]; that was 0.15 g/kg/day (maximum 9 g/day) [36] or 0.5 g/per meal [38], accordingly. The duration of intervention varied from 48 h to 2 months.

With regard to the primary outcomes (i.e., improvements in number, frequency, or severity of abdominal pain episodes), the effect of a low-FODMAP diet on abdominal pain intensity was reported in five included trials [36,37,38,39,40], pain frequency in two studies [37,40], and number of abdominal pain episodes in two studies [36,39]. A positive effect of a low-FODMAP diet on the primary outcomes was found in 4/5 trials [36,37,38,39], but only 2 trials [36,38] made between-group comparisons, whereas no effect was reported in 1 trial [40]. In terms of secondary outcomes, no effects of the low-FODMAP diet were observed on stool consistence. GI symptoms were reported in four trials [37,38,39,42] using various tools, of which half reported significant effects [38,39] and half reported no effects of the low-FODMAP diet on GI symptoms [37,42] compared to baseline or the control group. One study [39] reported less interference with daily activities after following a low-FODMAP diet compared to baseline values. Health-related quality of life was not reported in any study. The reported adherence to the low-FODMAP diet across the studies was 80% to 100%. The main characteristics of the studies are shown in Table 1.

Specifically, in a small open-label pilot study [37], researchers evaluated the effects of a low-FODMAP diet in eight children with IBS. Pain frequency, pain severity, and pain-related interference with activities decreased significantly in all children while on the low-FODMAP diet compared to baseline. However, four children (50%), defined as responders, showed a more robust response to the diet (>50% decrease in abdominal pain frequency while on a low-FODMAP diet) [37]. In a double-blind, crossover randomized controlled trial (RCT) [36] conducted by the same research group, 33 children with IBS were randomly assigned to either a low-FODMAP diet or a typical American childhood diet for 48 h, with a 5-day washout period between the intervention diets. Adherence to both diets was assessed using dietary records and weigh-ins and was similar between the low-FODMAP and the control diet (85.2 ± 15% and 90.7 ± 10.8%, respectively) after 48 h. The authors demonstrated a lower number of abdominal pain episodes in children on a low-FODMAP diet compared to children on the typical American childhood diet and baseline. Both the pilot and the RCT studies suggested that baseline gut microbiome composition and microbial metabolic capacity may play a role in responsiveness to the diet. Responders in the RCT had a baseline microbiome composition enriched with taxa (such as Bacteroides, Ruminococcaceae, Faecalibacterium prausnitzii, and Dorea) known for their greater saccharolytic metabolic capacity compared to non-responders, who were uniquely enriched at baseline with the Turicibacter genus from the Turicibacteraceae family.

In another RCT [38], 60 children with IBS were randomly divided into 2 groups to follow either a low-FODMAP diet or a standard gastrointestinal tract protective diet (i.e., defined as a diet that provides age-appropriate protein, calorie, vitamin, and mineral intake) for 2 months. Children were also reassessed for their symptoms and clinical status 2 months after the discontinuation of the intervention (4 months from baseline). Abdominal pain was evaluated using the Visual Analogue Scale (VAS), while the clinical status of each patient was assessed by their doctor using the Clinical Global Impression Improvement (CGI-I) scale. The authors claimed that the adherence to the dietary treatment was complete within two months, although no specific assessment tool was reported. After intervention, both VAS and GGI-I improved significantly in the low-FODMAP group compared to the control group. However, 2 months after the discontinuation of the intervention, both VAS and GGI-I were worse in the low-FODMAP group compared to the control group, suggesting that benefits from the adherence to a low-FODMAP diet are not sustained in the long term in children with IBS.

As far as FAP is concerned, a double-blind RCT evaluated the efficacy of the low-FODMAP diet in improving GI symptoms in 27 children with FAP diagnosed using Rome III criteria [40]. Patients were allocated to a low-FODMAP diet or a control diet based on the National Institute for Health and Care Excellence (NICE) guidelines for 4 weeks. Daily leftovers and times of self-reported noncompliance were assessed for evaluating adherence to the diets. At the end of the study, there were no between-groups significant changes in the abdominal pain intensity and frequency or in stool frequency and consistency. Higher noncompliance with the low-FODMAP diet was observed during the second week compared to the other weeks of the intervention within the low-FODMAP group compared to NICE group. There are no significant differences in the average percentage amount of daily leftovers in any week between groups.

The efficacy of a low-FODMAP diet for several GI outcomes in children with various FGIDs (FAP, IBS, or FD) has been explored in an open-label prospective study [39]. Abdominal pain (number and severity of episodes per week), interference with daily activities, stool characteristics, and associated symptoms, such as abdominal distension, gas, vomiting, and nausea, were evaluated using appropriate scales and questionnaires/records. The degree of adherence to the diet was self-reported based on a 5-point Likert scale questionnaire. Although patients were analyzed as a united group without differentiating each FGID, researchers reported improvements in abdominal pain episodes, abdominal pain intensity, and GI symptoms in the participants, as well as in interference with daily activities, after 2 weeks of the dietary intervention. Moreover, 13/20 reported substantial adherence, 6/20 a good adherence, and 1/10 fair adherence to the low-FODMAP diet.

The efficacy of a low-FODMAP diet for GI symptoms has been also evaluated in children with autism spectrum disorder (ASD) who have FC and/or abdominal pain [42]. Through a pilot single-site RCT [42], researchers concluded that a low-FODMAP diet for 2 weeks was effective in reducing not only the rates of constipation but also other GI problems (such as stomach pain and hurt, gas and bloating, stomach discomfort when eating, nausea and vomiting, and diarrhea) in children with ASD compared to their habitual diet. However, these improvements in GI symptoms did not connect to behavioral improvements in the participants. Of note, the low-FODMAP diet did not impact the nutrient intake of children’s diet, although the dietary adequacy at baseline was insufficient due to the related food selectivity, picky eating, and sensory problems commonly found in this population. The degree of adherence to the low-FODMAP diet was not reported by the authors.

Overall, the studies assessing the efficacy of a low-FODMAP diet with regard to the number of pain episodes and frequency or severity of abdominal pain or other GI symptoms in children with FGIDs are insufficient to support any therapeutic recommendations. Evidence is of low quality, due to small sample sizes, with a few studies being RCTs, whereas the tools used to assess GI outcomes varied across studies, which limits the uniform evaluation of the published results. Nevertheless, concerning specific FGIDs, a low-FODMAP diet may offer some benefit in selected children with IBS.

### 3.2. Fructose-Restricted Diet

Fructose and lactose malabsorption are considered as possible causes of recurrent abdominal pain (RAP) [45]. Lactose malabsorption (LM) is a frequent clinical condition caused by lactase-reduced activity (i.e., hypolactasia). The undigested lactose is fermented by the colonic flora, causing digestive symptoms. Fructose malabsorption (FM) is caused by the insufficient absorption and subsequent bacterial fermentation of fructose in the intestinal lumen [58].

Worldwide prevalence of LM is estimated to be 68%, with varied rates between countries [59]. However, only a small percentage of people seem to be lactose-intolerant (LI) [60]. The same is true for FM, as only a small percentage of children and adults present with symptoms after fructose ingestion (fructose intolerance, FI). Symptoms that are usually caused after lactose or fructose ingestion include flatulence, diarrhea, abdominal pain, and abdominal distension, symptoms similar to patients with FGIDs [61]. The likelihood of developing symptoms after fructose or lactose ingestion is multifactorial and seems to depend on the lactose/fructose dose, lactase expression, and the intestinal microbiome [60]. Hydrogen breath tests (HBTs) remain the most popular diagnostic method for assessing these conditions [62], although they often yield false-negative results in children [61]. Nevertheless, it has been found that children with visceral hypersensitivity associated with IBS and FAP may have LI/FI [63,64]. Consequently, in clinical practice, FRDs or LRDs are being proposed as less restrictive diets for the management of FGIDs, given that a low-FODMAP diet could compromise nutritional adequacy and lead to poor eating behavior in children [65].

In total, five studies were included in the present analysis: three evaluated the role of an FRD [28,43,44], one evaluated the role of an LRD [46], and one assessed the role of both FRD and LRD [45] in improving GI outcomes in patients with FGIDs. Three studies [28,43,45] included children with RAP, one study [44] enrolled patients with chronic abdominal pain, and one study [46] was on IBS patients. Two studies were uncontrolled clinical trials [43,44] two were RCTs [28,46], and one was a randomized placebo-controlled trial [45]. The latter [45] was the only study that reported the exact amount (i.e., 25 g of fructose and lactose with 2 g of glucose) allowed in the FRD and LRD trials. The duration of intervention varied from 2 weeks to 6 months.

With regard to the primary outcomes, abdominal pain severity was reported in three studies [28,43,46], whereas pain frequency was reported in two studies [28,43]. No studies were found evaluating the number of abdominal pain episodes. A positive effect of an FRD on primary outcomes was found in two trials [28,43] compared to baseline, whereas a positive effect of the LRD on primary outcomes was shown in one study [46] compared to the control group. With regard to secondary outcomes, stool frequency and missed school days per week were reported in one study [43], in which a positive effect of an FRD was reported compared to baseline values. GI symptoms improvements after following an FRD were found in two studies [43,44] compared to baseline values, whereas results from the randomized placebo-controlled trial [45] showed no effect of either an FRD or LRD on GI symptoms. The adherence to the FRD/LFD across the studies based on provided data was >80%. The characteristics of the studies are shown in Table 2.

In a single-arm clinical trial [43], 75 children with RAP for more than 3 months and positive fructose HBTs received an FRD for 4 weeks. The FRD group received detailed dietary advice with a list of allowed and disallowed foods and an option to call a dietitian in case of questions (no exact fructose dosage reported). A questionnaire asking for clinical symptoms, such as the intensity of pain and GI symptoms, and other parameters such as self-reported adherence to the diet was used. In total, 80% of patients declared adherence to FRD for more than 3 weeks and 88 % for more than 2 weeks. The authors reported that both pain frequency per week (1 vs. 4, *p* < 0.001) and intensity of pain (3 vs. 6, *p* < 0.001) as expressed by median changes reduced compared to baseline. Daily stool frequency, nausea, problems falling asleep, and missed school days also improved significantly (all *p* < 0.05).

The same research team, 2 years later, conducted a two-site prospective blinded RCT [28]. In total, 116 children with RAP for more than 3 months and positive fructose HBTs were placed to either an FRD or a regular diet (RD) for 2 weeks. What was new in this study, apart from the control group, was that children with positive fructose HBTs within the FRD group continued the FRD for 2 additional weeks at the end of the initial intervention (4 weeks from baseline). All subjects in the FRD group were advised to reduce fructose intake through dietitian-led counselling. No exact dosage of fructose was reported by the authors. Abdominal pain intensity, changes in pain frequency, and a secondary symptom score (SSS) assessing life quality parameters were evaluated through appropriate questionnaires. Adherence to the dietary intervention was self-reported by the participants at the 2-week follow up. Unfortunately, no between-groups comparisons were provided by the authors. At the end of 2 weeks, abdominal pain intensity was reduced within the FRD group (*p* < 0.001) compared to baseline. Interestingly, in both children with positive and children with negative fructose HBTs, abdominal pain improved within this group at 4 weeks. No changes were observed in the RD group. Both groups showed reduced pain frequency (74% vs. 57 %) compared to baseline. SSS also improved from 6 to 3.5 (*p* < 0.002) in the FRD group, with children with negative fructose HBTs showing significant improvement in SSS (*p* < 0.004) compared to baseline. No statistical changes were found within the control group in the SSS. No data were reported regarding the adherence to the FRD, although it was assessed by the authors.

Similarly, Escobar et al. [44] carried out a single-arm clinical trial that aimed to assess the role of an FRD in resolving GI symptoms of children with chronic abdominal pain. A total of 222 patients were included in the study, of whom 121 (54.5%) had positive and 101 (45.5%) had negative BHTs for fructose intolerance. The 121 patients with positive fructose HBTs received an FRD for 2 months. Adherence to the diet was self-reported. At the end of the study, 93/121 patients (76.9%) reported resolution of GI symptoms with FRD (*p* < 0.0001). All of these patients reported near-universal adherence to the dietary restrictions. Nevertheless, 55/101 patients (54.4%) with negative F-BHT also reported resolution of symptoms without an FRD, although the results did not reach significance (*p* = 0.37).

In contrast to previously reported studies, Gijsbers et al. [45] failed to prove any causal relationship between resolution of GI symptoms and FRD or LRD after conducting a randomized double-blind placebo-controlled trial [45]. Initially, 210 children with RAP were investigated for LM/LI or FM/FI. Of these, 57 (27%) were found to have positive lactose HBT and 79 (65%) positive fructose HBT. After assessing all patients through an elimination phase followed by an open provocation phase, six children with LM/LI and eight children with FM/FI were eligible for a randomized double-blind placebo-controlled trial in order to assess the role of an FRD or LRD in resolving GI symptoms in patients with RAP. At the end of the study, all patients in both groups tested negative, although GI symptoms continued. Patients showed improvements in their symptoms only in the elimination phase of the study. No information was given about the adherence to the FRD or LRD.

With regard to LI/LM, Gremse et al. [46] conducted a double-blind crossover RCT in children with IBS and positive lactose HBTs, aiming to prove a causal association between lactose ingestion and GI symptoms. In total, 33 patients received either 240 mL of lactose-hydrolyzed milk along with a lactose-free diet (LFD) or lactose-containing milk for 2 weeks. Adherence to the LFD was assessed through food diaries in order to evaluate possible sources of lactose ingested. At the end of the study, abdominal pain decreased in the LFD group compared to the control group (4.1 ± 1.4 vs. 7.5 ± 2.7, *p* = 0.021). Nevertheless, although 23/30 reported more symptoms after ingesting lactose, there were 7/30 who reported fewer or no symptoms, although they were fully compliant with LFD based on the evaluation of the food diaries.

Overall, the effective role of FRD or LRD in GI outcomes in children and adolescents with FGIDs (mainly in patients with RAP and IBS) is not clear. The coexistence and clinical importance of LI/LM or FI/FM in children with FGIDs remains unanswered. Current data depend on uncontrolled clinical trials or RCTs using separate paired tests against baseline, which possibly causes misleading conclusions [66]. Some patients with RAP or IBS may benefit from dietary fructose or lactose restriction, but the degree of the restriction or the amount of fructose or lactose that is allowed to be consumed without causing GI discomfort is poorly defined. Therefore, more data are required to draw strict conclusions on the beneficial effects (if any) of FRD or LRD in children and adolescents with FGIDs.

### 3.3. Gluten-Free Diet

Lately, there has been renewed interest in the role of gluten sensitivity as a potential trigger of GI symptoms in adults with IBS [67,68]. There are some RCTs [69,70] suggesting that adult patients with IBS may have intestinal (e.g., bloating and abdominal pain) and extra-intestinal symptoms (e.g., headache, anxiety, fibromyalgia-like syndrome, and skin rash) subsequent to the ingestion, despite a lack of celiac disease or wheat allergy [71,72]. This clinical condition is known as non-celiac gluten or wheat sensitivity (NCGS), although the term NCGS remain debatable, as it is unclear if gluten is the only wheat component to cause development of the GI symptoms [73]. NCGS may be present in children, with an estimated prevalence under 6%, although the true prevalence is difficult to determine [74]. This is because no specific diagnostic markers for NCGS or standardized diagnostic procedures exist, and therefore, the NCGS diagnosis usually requires observed, double-blind, placebo-controlled provocation testing [75].

Currently, three trials have been published investigating the effects of the GFD on FGIDs in the pediatric population [47,48,49]. Two studies were RCTs [48,49], and one was a double-blind placebo-controlled clinical trial [47]. Two studies [44,47] assessed the role of GFD in GI outcomes in children with several FGIDs, and one study [48] was on children with FAP/FC. The duration of intervention varied from 48 h to 2 months.

Only one study evaluated the changes in the abdominal pain severity after following a GFD compared to placebo (10 g of gluten challenge), showing no effects. Regarding our secondary outcomes, no effects of the GFD on GI symptoms were noticed in two studies compared to controls [47,48], whereas significant effects were noticed in one study compared to baseline values [49]. No reports were made regarding stool consistency or QoL. The reported adherence to the GFD was 80–91%. The main characteristics of the studies are shown in Table 3.

In a randomized double-blind placebo-controlled crossover trial [47], researchers screened 1114 children with chronic functional GI symptoms (i.e., chronic abdominal pain, diarrhea, bloating, dyspeptic symptoms diagnosed based on Rome III criteria) with or without extra-intestinal manifestations. Of these, 1078 (96.7%) did not present any correlation of symptoms with gluten ingestion and were excluded. The remaining 36 children followed a 3-phase trial: (a) run-in phase—2 weeks of exposure to a gluten-containing diet for baseline evaluation—in which 5 children presented with an improvement in symptoms (global VAS < 3) and were excluded; (b) an open GFD phase—2 weeks of gluten elimination, after which 31 continued, whereas 3 did not respond and were excluded from the next phase; and (c) a placebo-controlled crossover trial after 1 week of washout from the GFD, into which 28 children entered. All children received sachets (one per day) either with a placebo or with gluten (10 g of gluten plus 0.9 amylase/trypsin inhibitors (ATIs)). Adherence to intervention was evaluated through interviews and was calculated by the percentage of returned and ingested sachets in both groups. Based on the Salerno criteria (global VAS variation >30% between the gluten and the placebo challenge groups), 11 children (39.2%; 95% CI: 23.6–53.6%) tested positive, suggesting that 1 in 100 who were referred for chronic GI symptoms had NCGS. However, no differences were observed in global VAS and IBS severity scores or in clinical and biochemical characteristics of children when comparing challenges with gluten to placebo. Although the defined accepted adherence to the intervention was >80%, no exact percentage was reported at the end of the *p*. However, authors reported that participants were highly motivated, while there were no drop outs.

The GFD (and/or casein-free diet) has been also tested in children with ASD as a possible therapeutic approach, based on the hypothesis that the elimination of the peptides derived from the metabolism of gluten and casein may ameliorate behavioral and GI symptoms in this population [48].

In an RCT [49], 80 children with ASD were randomly subdivided into a GFD or a regular diet (RD) group for 6 weeks. In total, 38 children in each group completed the study. At baseline, 55.3% of the GFD group and 52.6% of the RD group had GI symptoms (e.g., stomachache, bloating, constipation, diarrhea) as diagnosed using Rome III criteria. At the end of the study, the prevalence of GI symptoms decreased significantly (40.57% vs. 17.10%, *p* < 0.05) within the GFD group, but no statistical changes were observed within the RD group (42.45% vs. 44.05%, *p* > 0.05). Behavioral improvements were also noticed within the GFD group (80.03 ± 14.07 vs. 75.82 ± 15.37, *p* < 0.05) but not in the RD group (79.92 ± 15.49 vs. 80.92 ± 16.24, *p* > 0.05). Unfortunately, researchers did not publish any between-group comparisons. No evaluation of the adherence to the diet was performed.

In contrast to the previous published study, in a single-blinded RCT [48], Piwowarczyk et al. aimed to determine whether a GFD compared to a gluten-containing diet (GD) could influence autistic symptoms, maladaptive behaviors, intellectual abilities, and GI symptoms in children with ASD after a 6-month intervention. Only abdominal pain and constipation were reported by the participants based on Rome III criteria. Autism symptoms, children’s adaptive capabilities, and cognitive abilities were evaluated through appropriate questionnaires. Adherence to the intervention diets was assessed through evaluating the presence or the absence of gluten, accordingly, in patients’ food records. After 8 weeks of an run-in GFD period, the GFD group continued this diet, and the GD group consumed at least one normal meal containing gluten per day for 6 months. Overall, researchers did not reveal any significant differences in autistic symptoms, maladaptive behaviors, intellectual abilities, or GI symptoms after the intervention between groups. Participants were compliant with the GFD by 91% and 85% in GD at the 12-week follow-up.

Overall, intervention data on the role of GFD in the treatment of FGIDs in pediatrics are scarce. Current evidence from intervention studies do not support the use of the GFD for the treatment of patients with FGIDs. More RCTs are needed to explore the efficacy (if any) of GFD in selected children with FGIDs.

### 3.4. Mediterranean Diet

The MD is primarily a plant-based dietary pattern characterized by high consumption of whole grains, fruits, vegetables, legumes, nuts, and seeds, as well as moderate amounts of dairy products and fish. Red meat and meat products are consumed in low quantities, while olive oil represents the main source of fat [76]. Robust evidence, based on meta-analyses of prospective cohort studies and RCTs, has proven the beneficial role of a greater adherence to the MD in a reduced risk of overall mortality, cardiovascular diseases, cancer incidence, neurodegenerative diseases, obesity, and diabetes [77]. The underlying mechanisms mediating the health benefits of the MD in health are attributable to the high intake of several bioactive compounds found in the MD, such as fiber, polyphenols, flavonoids, and monounsaturated and polyunsaturated fatty acids [78].

With regard to FGIDs, data coming mainly from epidemiological studies in adults [79,80] support that a higher adherence to the MD is associated with a lower prevalence and lower odds of having FGIDs compared to low adherence. This further suggests that MD could play a preventive role in the development of GI symptoms in those patients. However, limited evidence exists on the association between the MD and FGIDs in pediatrics [30]. Two epidemiological studies [9,65] have explored this association, along with the recently published results from our research team [9]. These studies confirmed what is already known from the adult population: good adherence to the MD is associated with a significant lower prevalence of FGIDs in both children and adolescents.

Clinical trials evaluating the efficacy of the MD in children and adolescents with FGIDs are lacking. Only one relevant open-label RCT [50] was found in the present review. Researchers subdivided 100 patients with IBS (diagnosed based on Rome IV criteria) into an MD group (with a good adherence to the MD, defined as KIDMED score ≥ 8 points), or a regular diet group for 6 months. A 100-point scale VAS total score was used to evaluate IBS symptoms, the IBS-symptoms-severity-score questionnaire (IBS-SSS) to assess the severity of IBS symptoms, and the IBS-QoL questionnaire (IBS-QoL) to evaluate patients’ QoL. MD was well tolerated by the patients without any adverse events. At the end of the study, within the MD group, the IBS-SSS and IBS-QoL scores improved compared to baseline, with no statistical changes in the regular diet group. Comparisons between groups at the end of the study also showed that IBS patients in the MD group compared to the regular diet group had lower total scores on IBS symptoms (*p* < 0.001), lower IBS-SSS (*p* < 0.001), and higher IBS-QoL scores (*p* = 0.03) [50].

Overall, the MD seems to be promising as a therapeutic approach in patients with FGIDs, especially for patients with IBS. Although results come literally from one RCT, the MD seems to be a well-known and tolerated dietary pattern that does not cause any adverse events in patients. However, future well-designed clinical trials are needed to verify current data.

## 4. Discussion

### 4.1. Summary of the Primary and Secondary Outcomes

In the present study, we systematically reviewed 15 clinical trials (both RCTs and non-randomized single-arm clinical trials) to determine the efficacy of specific dietary patterns, namely a low-FODMAP diet [36,37,38,39,40,41,42], an FRD or LRD [28,43,44,45,46], a gluten-free diet [47,48,49], and the Mediterranean diet [50], in the treatment for children with FGIDs. We concluded that no high-quality intervention trials exist, as the current evidence, according to the tools used to assess the risk of bias (i.e., ROB2 and ROBINS-I), was low (raises some concerns) to very low (serious concerns).

Bearing in mind these limitations, we found that there is insufficient evidence to support the use of a low-FODMAP diet or an FRD/LRD in children and adolescents with FGIDs. However, these dietary plans may offer some benefit in alleviating abdominal pain in some children with IBS or RAP. Moreover, the GFD should not be recommended for improving abdominal pain in children and adolescents in FGIDs, as current studies show no effects. The MD seems to be promising as a therapeutic approach in patients with IBS, although results come literally from one study. Overall, current evidence does not offer a robust background for drawing firm recommendations on specific dietary patterns that children and adolescents with FGIDs could follow in order to improve their symptoms or other GI outcomes. Future well-designed intervention studies are needed before transferring any of the available data into clinical practice.

With regard to secondary outcomes, no effects of the low-FODMAP diet were shown on stool consistency in children with FGIDs. However, Chumpitazi et al. [36,37] suggested that baseline gut microbiome composition and microbial metabolic capacity may play a role in responsiveness to the diet. Less interference with daily activities was also found while children were on a low-FODMAP diet. Mixed results were reported in terms of the efficacy of the FRD in improving GI and stool consistency in children with FGIDs. Moreover, results from RCTs show no effect of the GFD on GI symptoms, although improvements were seen when compared to baseline values. A positive effect of the MD was reported on IBS symptoms and QoL in patients with IBS compared to controls. Finally, researchers reported a substantial to good adherence of participants to most dietary patterns (i.e., more than 80% adherence was reported in every study) [36,37,38,39,40,43,47].

### 4.2. Literature Documention

In accordance with the present study, one previous relevant systematic review of RCTs [81] concluded that there are several methodological limitations of the available clinical trials on the efficacy of using a low-FODMAP diet in the treatment of children with FGIDs. Researchers concluded that the choice of the comparator diet (usually a non-standardized treatment for children with FGIDs compared to a placebo diet that is considered the gold standard method) as well as other domains (e.g., the success of blinding after follow-up, the carryover effects in crossover studies, the optimal duration of intervention) carried with them a high risk of bias [81].

In the present study, assessing the role of not only a low-FODMAP diet but also other dietary patterns in children with FGIDs, the current evidence was found to be low to very low e.g., only 2 randomized double-blind placebo-controlled trials were found (1 on GFD and 1 on FRD); a regular diet as a control diet was used only in 4 of the included controlled studies [28,42,49,50]; 2/3 crossover RCTs had “some concerns” arising from period and carryover effects). Furthermore, only five of the eligible studies [38,40,43,44,49] were in agreement with the recommendations published by the Rome Foundation for the appropriate intervention period (i.e., 4 weeks and preferably 6 weeks or more) when conducting a clinical trial in such patients. Most studies had rather too short (≤2 weeks) or too long (i.e., 6 months) intervention periods, which could further limit the addressing of the intended outcomes.

Currently, most of the available reviews in the literature assessing the efficacy of dietary interventions in treating patients with FGIDs have focused on the role of the low-FODMAP diet [57,82,83]; the use of dietary supplements, e.g., fiber [84], probiotics, prebiotics, or synbiotics [85,86,87]; or vitamin D supplementation [71]. Alternatively, they are not focused in pediatrics per se [88,89]. Although it is not within the purposes of the present review, fiber supplementation has been found to have a positive effect in the management of FGIDs in children [84], although for specific FGIDs (e.g., FC), their use is not recommended [90]. Nevertheless, the most frequent dietary recommendations given to children and adolescents with FGIDs in tertiary care or primary care are the use of fiber supplementation and a low-FODMAP diet [91]. Given that improvements in diet are considered as a first-line approach for the management of several diseases [92,93], there is a need for better justification of the dietary patterns that could be used in FGIDs in pediatrics.

In consistency with the present review, the position paper published in 2022 by the European Society for Paediatric Gastroenterology, Hepatology, and Nutrition (ESPGHAN) [57] suggested that there is insufficient evidence to recommend the use of the low-FODMAP diet for the treatment of FGIDs in children, apart from some patients with IBS. Currently, FRD or LRD are being proposed in clinical practice as less restrictive diets [48], but as shown by the present study, only some RAP or IBS patients may benefit from fructose restriction. Nevertheless, a major problem with the studies assessing the effects of a low-FODMAP diet or an FRD/FLD diet in patients with FGIDs is that the degree of the restriction, or the exact amount of FODMAPs/fructose/lactose that is allowed to be consumed without causing GI discomfort, is poorly defined. In the present review, only 2 studies reported the FODMAP content of the diet used: 0.15 g/kg/day (maximum 9 g/day) or less than 0.5 g/meal. In adults, differing FODMAP content has been tested depending on the diet used; allowed amounts have been 7.6 g/day, 15.2 g/day, and 22.4 g/day for a low-FODMAP diet, traditional dietary advice, and GFD, respectively. A suggested threshold for symptom improvements in adults is 12 g FODMAPs/day [29]. However, this has yet to be confirmed in pediatrics. Accordingly, in the only double-blind placebo-controlled diet evaluating an FRD/FLD in children with FGIDs, 25 g of either fructose or lactose was given, but several children continued to report abdominal symptoms upon fructose or lactose provocation.

We found no evidence of positive effects of GFD for the management of children with FGIDs. Currently, a strict gluten-free diet is a life-long necessity only for the treatment of patients with celiac disease [94]. Whether children and adolescents with IBS and NCGS could benefit from gluten elimination is not known. In one double-blind placebo-controlled study [47] in children with various FGIDs (included in the present review), gluten challenge with 10 g did not result in any GI improvements compared to placebo. However, gluten/placebo challenges in adults have shown mixed results [95]. For example, worse GI symptoms have been reported in adult patients with IBS and NCGS who were blindly exposed to gluten (68%), compared to the placebo group (40%) [69]. However, in another study, after a 2-week assignment to a low-FODMAP diet, different doses of gluten challenge (low, 2 g/d, vs. high, 16 g/d) did not cause any differences in GI symptoms in adult patients with IBS and NCGS [96].

We found little evidence for MD being effective for the treatment of FGIDs in children and adolescents. Although data come from one trial, two epidemiological studies in pediatrics [9,97] have explored this association, showing promising results. A cross-sectional study [9] conducted by our research team included 1972 children aged 4 to 9 years old and 2450 subjects aged 10 to 18 years old from 6 Mediterranean countries (i.e., Croatia, Greece, Israel, Italy, North Macedonia, and Serbia). The study aimed to reveal the possible associations of participants’ FODMAPS intake or adherence to the MD with the odds of having FGIDs. Higher compliance with the MD (as assessed by KIDMED score) was associated with lower odds of having FGIDs. In specific, each 1-unit increase in the KIDMED score was associated with a 17% lower possibility of having FGIDs in children aged 4 to 9 years old and a 7% lower possibility in children aged 10 to 18 years old [9]. A significant association was also found between the MD and FC as well as postprandial distress syndrome in both age groups. However, this was not the case with the FODMAP diet, as no significant associations were found between FGIDs and FODMAPs, in either age group. Agakidis et al. [97], through a prospective cohort study of 1116 children and adolescents, also showed similar associations. For each 1-unit increase in the KIDMED score, there was a 8.9% lower likelihood of having FGIDs after adjusting for age.

Finally, as evident in the literature, the prescription of a specific dietary pattern in children (as in adults) should involve a specialized pediatric dietitian in order to explain and supervise the adherence to the diet, a parameter that could also affect the potential outcomes of a study [57,98]. However, out of all children and adolescents with FGIDs who are provided with dietary recommendations in clinical practice, only 20% seem to receive an educational consult by a dietitian [91]. In this systematic review, most studies reported the involvement of a dietitian in their protocol, with the researchers suggesting a substantial to good adherence of participants to most dietary patterns (i.e., more than 80% reported in every study) [36,37,38,39,40,43,47]. However, a subsequent number of studies did not report any data [28,42,45,49]. The most important problem was the evaluation of the adherence to study diets, as in most cases it was self-reported without using any specific biomarker (e.g., determination of gluten immunogenic peptides (GIP) in stool or urine in order to verify gluten intake). In a recent literature review [99] assessing the adherence rates to dietary interventions in FGID patients, the reported range of adherence was 30–100%, with the most common method to measure adherence being food diaries. However, only one study in pediatrics was included, and data were mainly derived from studies implementing a low-FODMAP diet.

### 4.3. Strengths and Limitations

This systematic review has some limitations and some important strengths. The limitations of the present study are mostly associated with the low and very low quality of the studies included (most studies were characterized as having “some concerns” or “serious concerns”). Indeed, in order to increase the number of relevant studies identified (so as to improve the reliability of the study outcomes), we included non-randomized, single-arm clinical trials, which are generally considered intrinsically unsuited to demonstrating the benefit of a new treatment without the presence of a control group. We tried to address these issues by using up-to-date tools (i.e., Cochrane ROB2 and ROBINS-I) for assessing the risk of bias in the included studies. However, in some sections (e.g., MD), only one study was reported due to limited available data. Moreover, only three out of fifteen studies used the latest published criteria for the diagnosis of FGIDs, i.e., ROME IV criteria (most of trials used the ROME III criteria), and this is something that should be taken into consideration when interpreting the current study findings. Therefore, more future well-designed intervention studies are needed to overcome all these limitations.

The most important strength of the present study is that the methodology used was based on the high-quality standards of the Preferred Reporting Items for Systematic Reviews and Meta-Analyses (PRISMA), whereas for the agreement of study selections, clearly designated steps based on the Rayyan tool were used.

## 5. Conclusions

In the present study, we systematically reviewed 15 clinical trials (RCTs, non-RCTs, and single-arm clinical trials) to determine the efficacy of specific dietary patterns (i.e., a low-FODMAP diet, an FRD or LRD, a GFD, and the MD) in the treatment of children with FGIDs. We demonstrated the lack of high-quality intervention trials. Based on the current evidence, low-FODMAP diet, LRD, FRD, and GFD do not have a place in daily practice for the management of children and adolescents with FGIDs. Nevertheless, some patients with IBS or RAP may have some benefit from the use of a low-FODMAP diet or an FRD/LRD. Limited data suggest that MD may be promising in treating FGIDs, especially in IBS patients, but more data are required to draw conclusions on its protective effects.

## Figures and Tables

**Table 1:** Characteristics of the studies assessing the role of a low-FODMAP diet in the treatment of functional gastrointestinal disorders in children and adolescents.

Author- Journal- Year of Publication	Type of Study	Sample	Diagnosis	Study Groups	Intervention	Duration	Follow-Up	Age	Outcomes	Tools Used	Study Results	Adherence to the Intervention
Chumpitazi, B.P. et al. Alimentary pharmacology & therapeutics 2015 [36]	double-blind, crossover RCT-5 days washout period between intervention diets	33	IBS (Rome III criteria)	low-FODMAP diet group (*n* = 16) or TACD (*n* = 17)	The low FODMAP diet contained 0.15 g/kg/day (maximum 9 g/day) of FODMAPs. The TACD contained 0.7 g/kg / day (maximum 50 g/day) of FODMAPs.	48 h		7–17 years	Pain episodes (i.e., abdominal pain location, severity and duration), associated daily GI symptoms, microbiome composition/metabolic capacity, gas production (hydrogen & methane)	7-day pain and stool diary before intervention and for the 2 days in each group, 0–10 Likert scale for GI symptoms, modified Bristol stool form chart for stool characterization. Adherence was based on food records and weight-ins.	Less abdominal pain episodes/day in the FODMAP diet vs. TACD [1.1 ± 0.2 vs. 1.7 ± 0.4, *p* < 0.05] and compared to baseline (1.4 ± 0.2) (*p* < 0.01) but more episodes during the TACD (*p* < 0.01).	90%
Chumpitazi, B.P. et al. Gut microbes 2014 [37]	open-label pilot study	12 (*n* = 8 completed the study)	IBS (Rome III criteria)	low FODMAP diet group (no control gorup)	Instructions to decrease high FODMAP foods, sample menus and a table detailing foods to avoid and foods allowed, option to contact dietitian	1 week		7–16 years	Abdominal pain severity & frequency, Stooling characteristics and transit time, gas production (hydrogen & methane), microbial communities and associated metabolites	7-day pain and stool diary, 3-day food record, stool for microbiome composition, validated 0–10 scale for measuring abdominal pain (0 = “no pain at all” and 10 = "worst pain you can imagine")	Pain frequency (*p* < 0.05), pain severity (*p* < 0.05), and pain-related interference with activities (*p* < 0.05) decreased in the subjects while on the low-FODMAP diet. Responders vs. non-responders: four children (50%) were identified as responders (>50% decrease in abdominal pain frequency while on the low-FODMAP diet). There were no differences between responders and non-responders with respect to hydrogen production, methane production, stooling characteristics, or gut transit time.	High adherence defined as increased consumption of low-FODMAP foods
Dogan, G. et al. Northern clinics of Istanbul 2020 [38]	RCT	60	IBS (Rome IV criteria)	low-FODMAP diet group (*n* = 30) or protective GI diet group (*n* = 30)	FODMAP intake was less than 0.5 g per meal. A healthy diet list was given to the control group.	2 months	2 months	6-18 years	Abdominal pain severity, abdominal distention, defecation habits, clinical status (i.e., abdominal pain, boalting and general well-being status of the patient) at the end of the study and after 2-month of follow-up (no intervention given)	10cm Visual Analogue Scale (VAS) for abdominal pain, Clinical Global Impression Improvement scale (CGI-I) for the assessement of clinical status by doctors. No specific tool was reported with regards to adherence to the diets.	Post intervention: Decrease in VAS score in low-FODMAP group vs. control group (3.80 ± 1.10 vs. 2.03 ± 1.03, *p* = 0.0001) and in CGI-I (*p* = 0.0001). At follow-up: increase in VAS score in low-FODMAP group vs. control group (2.97 ± 1.10 1.63 ± 0.71, *p* = 0.0001), but desease in CGI-I score (*p* = 0.0007).	100% adherence in 2 months
Baranguan Castro, M.L. et al. An Pediatr (Engl Ed) 2019 [39]	open-label prospective study	22 (*n* = 20 completed the study)	various FAPDs; FAP, IBS, or FD (Rome III criteria)	low FODMAP diet group (no control gorup)	A table of ‘‘allowed’’ or ‘‘not allowed’’ foods based on their FODMAP content was given.	2 weeks		5–15 years	Abdominal pain (number and severity per/w) Interference with daily activities, stools characteristics, associated symptoms, such as abdominal distension, gas, vomiting, nausea and other	10-cm Visual Analogue Scale (VAS) for abdominal pain intensity, 4-point Likert scale for the assessment of interference with daily activities, Bristol stool scale modified for children, 5-point Liker scale for assessing the degree of the adherence to the diet.	Less number of abdominal pain episodes per day compared to baseline [1.16, (0.41–3.33) vs.2, (1.33–6.33), *p* = 0.024], lower 10-cm VAS compared to baseline [1.41 (0.32–5.23) vs. 4.63 (2.51–6.39), *p* = 0.035], less interference with daily activities, fewer associated symptoms like abdominal distension or gas, no differences in stool charasteristics	13/20 substancial adherence, 6 good adherence,1 fair adherence
Boradyn, K.M. et al. Annals of Nutrition and Metabolism 2020 [40]	double-blind RCT	29 (27 completed the study)	FAP (Rome III criteria)	low-FODMAP diet (*n* = 13) vs. NICE (*n* = 14)	Pre-cooked meals prepared based on the food grading system proposed by the Monash University in the Low FODMAP Diet AppTM.	4 weeks		5–12 years	Abdominal pain (frequency & intensity), Stool consistency	Wong-Baker FACES Pain Rating Scale for pain severity. Daily leftovers and times of noncompliance were assessed for evaluating adherence to diets.	No between groups significant changes in the abdominal pain intensity and frequency as well as in stool frequency and consistency. No significant changes within low-FODMAP group but significant reduction in abdominal pain intensity and frequency (*p* < 0.01) and improvement in stool consistency (93% reporting normal stool, *p* < 0.05) in the NICE group.	Higher noncompliance to the diet was observed during the second week in the low FODMAP group compared to NICE group. No significant differences were seen in the average percentage amount of daily leftovers in any week between groups.
Nogay, N.H. et al. Journal of Autism and Developmental Disorders 2021 [42]	pilot single-site, *RCT*	15	ASD with constipation and/or abdominal pain (Rome IV criteria)	low FODMAP diet (*n* = 7) or control group (habitual diet, *n* = 8).	Detailed nutrition education by the investigator (Dietitian) concerning the low FODMAP diet	2 weeks		6–17 years	GI module total score, GI symptoms total score. Stool frequency and consistency. Behavioral problems	Dietary food record (3 days before start to study and the last 3 days of the study), stool consistency/frequency record (3 days before start to study and the last 3 days of the study). Aberrant Behavior Checklist-Community and Pediatric Quality of Life Inventory Gastrointestinal Module	Reduced rates of constipation, GI module and symptoms scores (*p* < 0.01) (i.e., reduced stomach pain and hurt, gas and bloating, stomach discomfort when eating, nausea and vomiting, and diarrhea) in the low-FODMAP group compared to the control group. No statistical significance in the stool frequency and consistency both in the low FODMAP diet and control groups compared to baseline.	NR

ASD = Autism Spectrum Disorder; CD = celiac disease; FAP = Functional Abdominal Pain; GIC = non-coeliac with mild chronic gastrointestinal complaints; GSS = GI Symptom Scale; HC = healthy controls; IBS = Irritable bowel syndrome; NICE = dietary recommendations from the National Institute for Health and Care Excellence; RCT = Randomized Clinical Trial; TACD = Traditional American Children diet; NR = not reported; VAS = Visual Analogue Scale.

**Table 2:** Characteristics of the studies assessing the efficacy of a fructose- or lactose-restricted diet in the treatment of functional gastrointestinal disorders in children.

Author- Journal- Year of Publication	Type of Study	Diagnosis	Study Groups and Sample	Intervention	Duration	Age	Outcomes	Tools Used	Evaluation	Study Results	Adherence to the Intervention
**Fructose Intolerance/Malabsorption**											
Wirth, S. et al. Klin Padiatr 2014 [28]	2 site-prospective, blinded RCT	Children with RAP for >3 months with positive F-HBT	*n* = 116 total sample/*n* = 103 completed the study. FRD (*n* = 51) or regular diet (RD) (*n* = 52)	FRD: detailed dietary counselling for fructose restriction plus 10 recipes for warm meals. Regular diet: instructions no to alter their diet	2 weeks (plus 2 additional weeks for children with positive F-HBT within FRD group).	3–18 years (3.4 to 16.4 years, *n* = 103)	abdominal pain intensity, changes of pain frequency, secondary symptom score (SSS) (range 0–24) 8 parameters evaluated: nausea, vomiting, fatigue, sleep disturbance, headache, dizziness, anorexia and use of pain relievers (Scores 0 to 3).	10-point Likert scale (0 = no pain, 10 = very strong pain) for pain intesity, 3-poin scale (0 = never, 3 = frequent) for SSS, pain frequency was recorded through questainnaire. Adherence to the diets was assessed through questionnaire at the 2-week follow up.	F-HBT with 1 g fructose/kg body weight and a maximum of 25 g in a 10% solution after 8h fasting.	**Abdominal pain intensity:** reduced in FRD (*p* < 0.001) but not in RD (*p* > 0.5). Within FRD, children with both positive and negative F-HBTs reduced abdominal pain. **Abdominal pain frequency:** both groups reduced pain frequency (74% vs. 57%). **SSS results:** FRD: SSS reduced from 6 to 3.5, *p* < 0.002, RD: no statistical change. Within FRD, children with negative F-HBTs reduced SSS (*p* < 0.004).	No data reported although adherence was assessed by the authors.
Wintermeyer, P. et al. Klin Padiatr 2012 [43]	single arm clinical trial	Children with RAP for the previous 3 months with positive F-HBT	*n* = 75 in FRD / no control group	FRD:detailed dietary advice with a list of allowed and not allowed foods were given / option to call dietitian in case of questions.	4 weeks	3–14 years	Frequency and intensity of abdominal pain, stool frequency per day, nausea, problems to fall asleep, missed school days per week, and use of pain relievers	A questionnaire asking participants for clinical symptoms, e.g. frequency of pain, pain intensity, GI symptoms and adherence to the diet was used. Pain intensity was assessed through a 10-point Likert scale questionnaire (0 = no pain, 10 = very strong pain)	F-HBT with 1 g fructose/ kg body weight with a maximum of 25 g in a 10% solution after an 8–12 h fasting period	At the end of the study, pain frequency/w (1 vs.4, *p* < 0.001) and the intensity of pain (3 vs.6, *p* < 0.001) reduced compared to baseline. Daily stool frequency, nausea, problems to fall asleep, missed school days also improved significantly (all *p* < 0.05).	80% of patients declared adherence to fructose restricted diet for more than 3 weeks and 88% for more than 2 weeks.
Escobar, J. et al. Gastroenterology 2014 [44]	single arm clinical trial	Children with chronic abdominal pain	121 of 222 patients (54.5%) with positive F-HBT were placed on FRD	1-hour individual consultation with a dietitian, a list of allowed and not allowed foods and a sample menu	2 months	2−19 years	Resolution of GI symptoms	A standard pain scale. Adherence to the diet was assessed through patient report.	F-HBT with 1 g/kg fructose to a maximum of 25 g after 12 h of fasting	At the end of the study, 93/121 patients (76.9%) reported resolution of GI with FRD (*p* < 0.0001). Moreover, 55/101 patients (54.4%) with negative F-BHT reported resolution of symptoms without a FRD (*p* = 0.37).	All patients with positive F-HBT reported near universal adherence to the dietary restrictions.
**Fructose or Lactose Intolerance/Malabsorption**											
Gijsbers, R. et al. Acta Paediatrica 2012 [45]	randomized double-blind placebo-controlled trial	Children with RAP and positive F-HBT or L-HBT	**LM/LI patients =>** initial screen phase: *n* = 210 with 57 positive L-HBT, elimination phase: *n* = 38/57 with 24 positive L-HBT. Open provocation phase: *n* = 23/24 with *n* = 7 positive L-HBT. DBPC phase: *n* = 6/7 with *n* = 6 negative L-HBT. **FM/FI patients =>** initial screen phase: *n* = 121 with 79 positive F-HBTs, elimination phase: *n* = 49/79 with *n* = 32 positive F-HBTs, provocation phase: *n* = 31/32 with *n* = 13 positive F-HBTs, DBPC phase: *n* = 8/13 with *n* = 8 negative F-HBTs.	DBPC: containers with 25 g lactose or fructose and 2 with glucose in amounts that resulted in the same sweetness, numbered 1 through 4 in a randomized way.	6 months	4.1–16.0 years [mean age 8.8]	Resolution of GI symptoms	Not defined	F-HBTs and L-HBTs of 2 g/kg, with a maximum of 50 g in a 16.7% (50 g/300 mL) solution	After the DBPC phase, all patients with positive F-HBTs or positive-HBTs tested negative. No causal relationship between DBPC and FAP was proven by researchers.	NR
**Lactose Intolerance/malabsoprtion**											
Gremse, D.A. et al. Clin Pediatrics, 2003 [46]	double-blind, crossover RCT	Children with IBS and positive L-HBT	Interventiong group: 240 mL of lactose-hydrolyzed milk or lactose-containing milk along with LRD (*n* = 33 in a crossover design)	Intervention group: Lactose-free milk prepared with 2.0 g of lactase per 1.9 L milk. Control group: lactose-containing milk +aspartame 1.5 g per 1.9 L of milk.	2 weeks	3–17 years	pain severity, total GI symptoms score	Food diaries were used to assess adherence to the LFD, pain diaries collected weekly, pain severity assesed by with a 4- likert scale (0, no symptoms; to 4, severe symptoms), total symptom score for each patient.	L-HBTs of 1 g/kg (up to 50 g) was given in a 10% solution after overning fasting	At the end of the study, abdominal pain severity decreased in the intervention compared to the control group (4.1 ± 1.4 vs. 7.5 ± 2.7, *p* = 0.021). Within the control group, 23/30 reported more symptoms. However, 7/30 reported less or no symptoms, although compliant with the diet.	Fully adherence to the LFD.

**Table 3:** Characteristics of the studies assessing the efficacy of the GFD for the treatment of functional gastrointestinal disorders in children.

Author- Journal- Year of Publication	Type of Study	Diagnosis	Phases and Sample	Intervention	Duration	Age	Outcomes	Tools Used	Study Results	Adherence to the Intervention
Piwowarczyk, A. et al. Journal of Autism and Developmental Disorders 2020 [48]	Single arm- blind RCT	Children with autism spectrum disorders (ASD) plus FAP and FC (Rome III criteria)	(a) 8-week run in period, (b) 6-month GFD (*n* = 28 of which 27 had FGIDs) or GD (*n* = 30 of which 29 had FGIDs)	GFD: no consumption of gluten, GD: at least one normal meal containing gluten per day	6 months	36–69 months	autistic symptoms, maladaptive behaviors, intellectual abilities and GI symptoms	ADOS-2 for autism symptoms, SCQ &ASRS for diagnosis of ASD, VABS-2 for child’s adaptive capabilities, Leiter International Performance Scale for participants’ cognitive abilities, Rome III, adherence to the diets was assessed through food records.	No significant results in autistic symptoms, maladaptive behaviors, intellectual abilities or GI symptoms after the intervention between GFD and CG groups (all *p* > 0.05).	91% in the GFD and 85% in GD at 12-week follow-up.
Francavilla, R. et al. Am J Gastroenterol 2018 [47]	Double-blind, placebo controlled crossover CT-1 week washout period	Children with a positive history of FGIDs (i.e., chronic abdominal pain, diarrhea, bloating, dyspeptic symptoms) with or without extra-intestinal manifestations (Rome III criteria)	(a) 2-week run-in period (*n* = 36), (b) 2-week open GFD (*n* = 31) (c) 2-week double-blind placebo-controlled crossover gluten challenge (*n* = 28)	Gluten (10 g/daily) and placebo (rice starch) given as placebo or gluten sachets (one per day)	2 weeks for each phase	11.4 ± 4.3 (GFD responsive)	pain severity, prevalence of NCGS, clinical and/or laboratory parameters at baseline, NCGS clinical profile	Global VAS, IBS-SS, STAIC, adherence was evaluated through interviews and was calculated by the percentage of returned and ingested sachets.	Eleven children (39.2%; 95% CI: 23.6–53.6%) tested positive for NCGS. No significant differences were observed in global VAS and IBS-SS as well as in clinical and biochemical characteristics of children when comparing challenges with gluten to placebo (all *p* > 0.05).	Not exact percentage was reported but the minimun accepted adherence was >80% with no drop outs.
Ghalichi, F. et al. World J of Pediatrics 2016 [49]	RCT	Children with autism spectrum disorders (ASD) plus part of them with FGIDs (stomachache, bloating, constipation, diarrhea) (Rome III criteria)	*n* = 38 in GFD (55.3% with FGIDs) and *n* = 38 in RD (52.6% with FGIDs)	GFD: no consumption of gluten, RD: regular diet	6 weeks	7.92 ± 3.37 (total sample)	GI symptoms, behavioral indices	Rome III, ADI-R, GARS-2	GFD: GI symptoms decreased (40.57% vs. 17.10%, *p* < 0.05) and behavioral tests improved (80.03 ± 14.07 vs. 75.82 ± 15.37, *p* < 0.05). RD: no statistical changes observed in GI symptoms or behavioral test. No between groups comparisons provided.	NR

ADI-R = Autism Diagnostic Interview-Revised; ADOS-2 = Autism Diagnostic Observation Schedule-2; ASRS = Autism Spectrum Rating Scale; FAP = functional abdominal pain; FC = functional constipation; GARS-2 = Gilliam Autism Rating Scale 2; GD = gluten diet; GFD = gluten-free diet; IBS–SS = Irritable bowel syndrome–severity score; NCGS = non-celiac gluten or wheat sensitivity; NR = not reported; RCT = Randomized Clinical Trial; RD = regular diet; STAIC = State-Trait Anxiety Inventory for Children; SCQ = Social Communication Questionnaire; VABS-2 = Vineland Adaptive Behavior Scale-2; VAS = visual analogue scale. CT = clinical trial; F-HBTs = Fructose-hydrogen breath tests; FRD = fructose restricted diet; IBS = Irritable bowel syndrome; L-HBTs = Lactose-hydrogen breath tests; LFD: lactose free diet; LRD = lactose restricted diet; NR = not reported; RAP = recurrent abdominal pain; SSS = secondary symptom score; RCT = Randomized Clinical Trial; RD = regular diet.

## Supplementary Materials

The following supporting information can be downloaded at https://www.mdpi.com/article/10.3390/nu15122708/s1. Figure S1: Risk of bias of the eligible non-RCTs/single-arm clinical trials using the Risk Of Bias In Non-randomized Studies—of Interventions (ROBINS-I) [32]. Figure S2: Risk of bias of the eligible RCTs with crossover design using the Cochrane Risk of Bias tool (ROB-2) for crossover trials [35]. Figure S3: Risk of bias of the eligible RCTs using the Cochrane Risk of Bias tool (ROB-2) [34].
