# Supplementary material for: Efficacy of Different Dietary Patterns in the Treatment of Functional Gastrointestinal Disorders in Children and Adolescents: A Systematic Review of Intervention Studies
Source: Nutrients. 2023 Jun 10;15(12):2708. doi: 10.3390/nu15122708 (PMC10302595; doi:10.3390/nu15122708)
Supplement: Supplementary file 1 [file nutrients-15-02708-s001.zip › nutrients-2410127-supplementary.pdf]

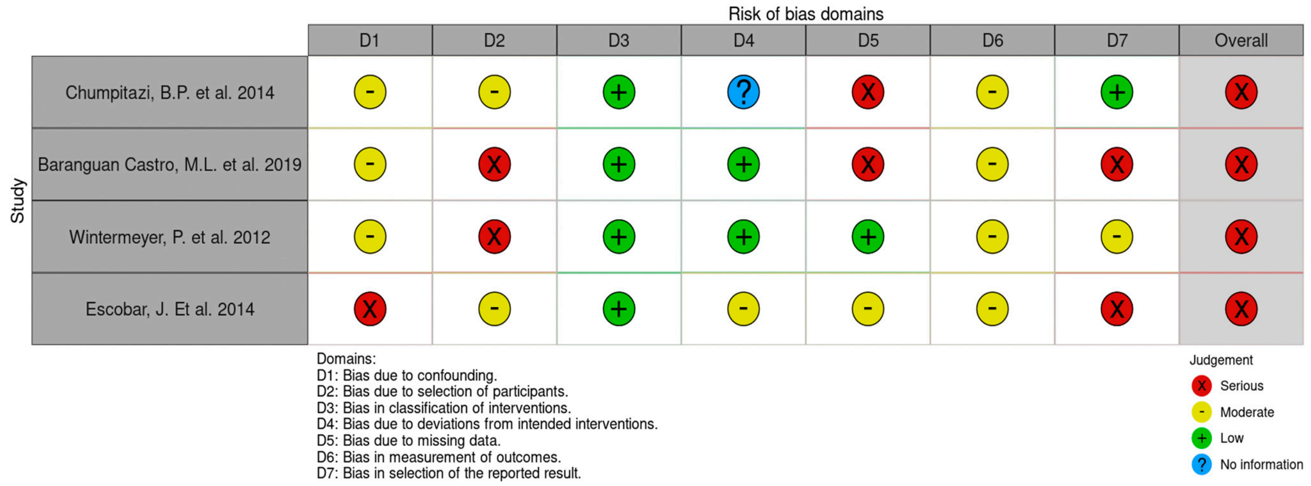

Figure S1. Risk of bias of the eligible non-RCTs/single arm clinical trials using the Risk Of Bias In Non-randomized Studies - of Interventions (ROBINS-I) [32] tool.

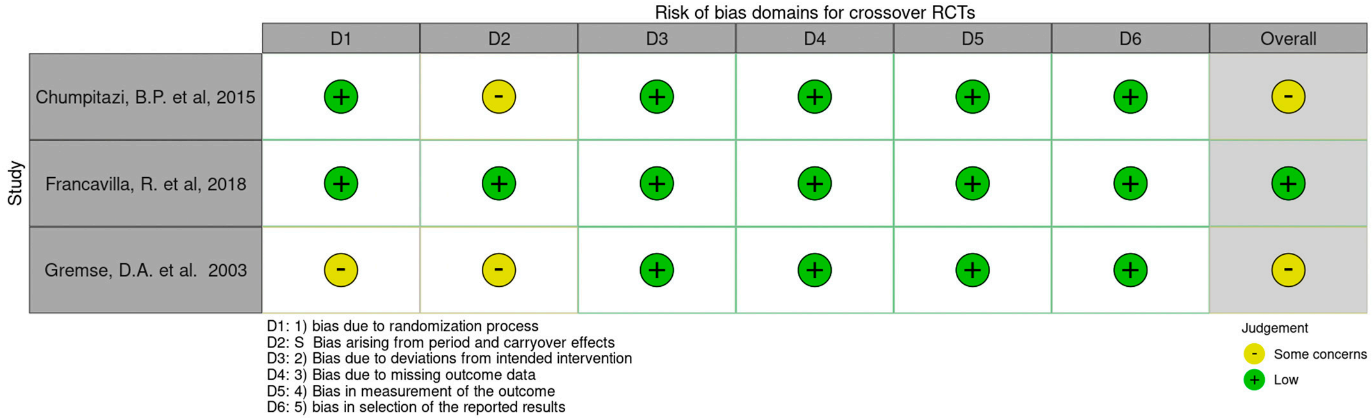

Figure S2. Risk of bias of the eligible RCTs with crossover design using the Cochrane Risk of Bias Tool (ROB-2) [35] tool for crossover trials.

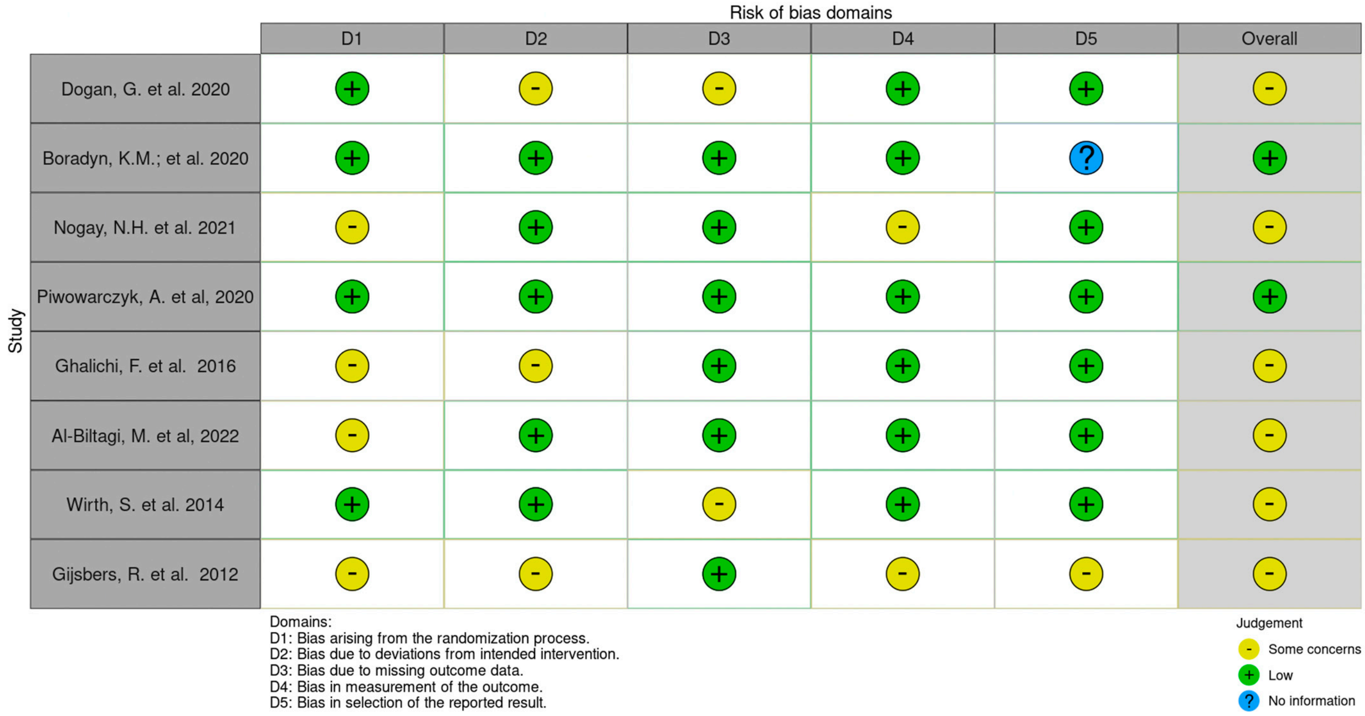

Figure S3. Risk of bias of the eligible RCTs using the Cochrane Risk of Bias Tool (ROB-2) [34].

References:

32. Sterne, J.A.; Hernán, M.A.; Reeves, B.C.; Savović, J.; Berkman, N.D.; Viswanathan, M.; Henry, D.; Altman, D.G.; Ansari, M.T.; Boutron, I. ROBINS-I: a tool for assessing risk of bias in non-randomised studies of interventions. bmj 2016, 355.

34. Sterne, J.A.; Savović, J.; Page, M.J.; Elbers, R.G.; Blencowe, N.S.; Boutron, I.; Cates, C.J.; Cheng, H.-Y.; Corbett, M.S.; Eldridge, S.M. RoB 2: a revised tool for assessing risk of bias in randomised trials. bmj 2019, 366.

35. Higgins, J.P.; Li, T.; Sterne, J. Revised Cochrane risk of bias tool for randomized trials (RoB 2): Additional considerations for crossover trials. Cochrane Methods 2021, 1-6.
